# Enhanced Cellular Materials through Multiscale, Variable-Section Inner Designs: Mechanical Attributes and Neural Network Modeling

**DOI:** 10.3390/ma15103581

**Published:** 2022-05-17

**Authors:** Nikolaos Karathanasopoulos, Dimitrios C. Rodopoulos

**Affiliations:** Department of Engineering, Abu Dhabi Campus, New York University, Abu Dhabi P.O. Box 129188, United Arab Emirates; d.rodopoulos@nyu.edu

**Keywords:** multiscale, metamaterials, machine learning, neural networks, experimental testing

## Abstract

In the current work, the mechanical response of multiscale cellular materials with hollow variable-section inner elements is analyzed, combining experimental, numerical and machine learning techniques. At first, the effect of multiscale designs on the macroscale material attributes is quantified as a function of their inner structure. To that scope, analytical, closed-form expressions for the axial and bending inner element-scale stiffness are elaborated. The multiscale metamaterial performance is numerically probed for variable-section, multiscale honeycomb, square and re-entrant star-shaped lattice architectures. It is observed that a substantial normal, bulk and shear specific stiffness increase can be achieved, which differs depending on the upper-scale lattice pattern. Subsequently, extended mechanical datasets are created for the training of machine learning models of the metamaterial performance. Thereupon, neural network (NN) architectures and modeling parameters that can robustly capture the multiscale material response are identified. It is demonstrated that rather low-numerical-cost NN models can assess the complete set of elastic properties with substantial accuracy, providing a direct link between the underlying design parameters and the macroscale metamaterial performance. Moreover, inverse, multi-objective engineering tasks become feasible. It is shown that unified machine-learning-based representation allows for the inverse identification of the inner multiscale structural topology and base material parameters that optimally meet multiple macroscale performance objectives, coupling the NN metamaterial models with genetic algorithm-based optimization schemes.

## 1. Introduction

Progress in additive manufacturing has allowed for the engineering of customized structures with highly refined inner designs, well below the micrometer scale [1]. Mechanical parts have been fabricated with characteristics that would have been infeasible using traditional manufacturing processes [2]. Moreover, a wide range of advanced materials—named metamaterials—have been developed, with tailorable effective mechanical properties [3,4,5] and design flexibility that has opened new frontiers in the control of the functional response of structural components [6,7,8].

Metamaterials have typically been based on the design of periodic inner structural patterns that can yield macroscopic mechanical properties with fundamentally different attributes from the ones observed for the base material used [9,10]. This quest has fostered the engineering of advanced materials with extraordinary mechanical behaviors such as the development of auxetics [11,12], and thus, materials which laterally expand instead of contracting upon the application of tensile loads [13,14,15,16]. The aforementioned non-conventional volumetric response has been primarily materialized by re-entrant patterns, with indicative examples being the star-shaped or the re-entrant honeycomb periodic cell designs [17,18,19].

Depending on the unit-cell architecture selection, different material performance objectives can be fulfilled, such as high specific stiffness to normal and shear loads, or high yield-strength limits for a target relative density design [10]. Certain periodic patterns have been associated with high specific elastic stiffness attributes—with indicative examples being the triangular and kagome lattice patterns [20,21]—while others are associated with a unique volumetric bulk response [22] or resistance to shape-changing (shear) loadings [23]. It should be noted that the resistance of an architected material to volumetric or shape changes is primarily determined by its unit-cell pattern and is typically considerably different from the one expected for the base material employed [24]. In particular, metamaterials with a substantially high or low bulk K and shear modulus G can be engineered upon selection of the appropriate lattice architecture, using common steel or aluminum alloys as the base material [23]. Interestingly, modifying the slenderness of the elements that comprise the basic unit-cell does not affect the relative bulk-to-shear (K/G) response of several lattice patterns, so that hexachiral or re-entrant star-shaped metamaterials with a relative density of 0.1 or 0.2 yield the same relative bulk-to-shear K/G response, even though each density design relates to a different bulk K and shear G resistance [24]. As a result, their relative volumetric-to-shape resistance performance remains invariant, even though their individual elastic, shear and relative density attributes are different [24].

An extended space of effective material attributes can be achieved through the introduction of structural hierarchies into the basic topological framework, leading to increased tensile elastic characteristics, with nearly-constant specific stiffness [25,26]. Hierarchical, multiscale constructions can not only materialize unique elastic mechanical properties that are infeasible by single-scale designs, but also modify the post-elastic [27], collapse load [28] and buckling resistance of the primal, single-scale metamaterial [29]. The manipulation of the macroscale effective material properties, achieved through the insertion of additional architectural scales, is lattice-specific, with the design at the additional inner scale to decisively modify the overall material performance, as illustrated for anti-tetrachiral, two-scale lattices [30].

The computation of the effective metamaterial attributes presupposes the development of models that can associate microscale designs with macroscale response metrics. With that, simplified analytical [31] and numerical homogenization models [32] have been developed, most of them applicable to single-scale material architecture [33]. It has to be noted that the extraction of the different constitutive elastic response parameters (e.g., normal, shear, and bulk moduli) requires the computation of a minimum of two independent loading test cases, for a unique inner-design architecture [34]. Moreover, an experimental extraction of the effective metamaterial attributes is typically limited by practical constraints related to the number of loading scenarios or inner designs that can be probed.

**Figure 1:**
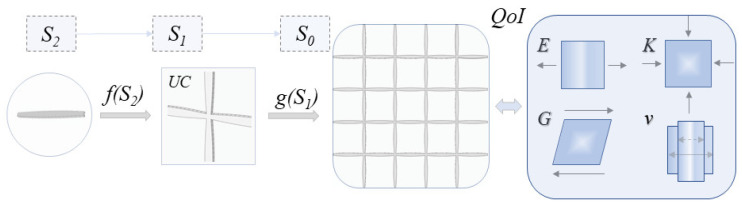
A multiscale metamaterial design, with the effective material properties (S_0_) to be determined by the first-scale unit-cell (UC, S_1_) and inner-element scale design (S_2_) of the periodic pattern. The effective metamaterial normal E, bulk K, shear G and Poisson’s ratio ν attributes are a function of the S_2_ and S_1_ scale design.

Recently, machine learning techniques have been employed for the computation of the effective constitutive response of foam materials [35], as well as for the characterization of functional 3D mechanical metamaterials [36]. Machine learning in material science has signified a transition to data-driven modeling approaches [37,38,39]. Models of the kind can reduce computing costs by several orders of magnitude, providing the possibility to relate a wide range of input design parameters with the desired quantities of interest (QoI, Figure 1) within a single computational step [40]. However, their successful training requires an appropriately designed machine learning model architecture [41], along with the existence of sufficient data for model training and validation to be feasible [42]. Until now, neural networks have been extensively employed to simulate structure-property-related functions [43,44,45], to identify functional relationships [46,47,48], as well as to optimize inner structural topologies [49,50,51,52,53,54]. Amongst others, spinodoid or curved inner beam architectures have been considered [55,56].

One of the major challenges in the development of high-performing machine learning methods of this kind is the generation of databases that are informative enough for the relevant solid mechanics response to be captured. Finite element models have been used to generate datasets for high-contrast [43,57] and tessellate composites [58]. Moreover, machine learning models have been developed to identify the relationship between salient structural features and the uniaxial compressive response of advanced materials. In particular, the geometric design of cellular materials has been used as an input for deep neural networks to be trained, so as to predict the macroscale uniaxial response of non-uniform cellular solids [59].

While it is currently well-established that the insertion of inner-material scales beyond the primal cellular pattern provides additional degrees of freedom for design, the corresponding material spaces remain, to a great extent, unexplored. Existing contributions have mainly emphasized on hollow inner designs [60,61], with the form of their inner structural components remaining unchanged. In particular, multiscale cellular metamaterials with hollow, variable-section inner designs have not been yet analyzed, their macroscale material performance remaining unquantified, both numerically and experimentally. Moreover, relevant data-based machine learning models that can associate multiple inner-scale input design parameters with the complete set of effective macroscale cellular material attributes have not been developed. Therefore, the modeling specifications and complexity required for such a task remain uncharacterized. Moreover, the potential use of surrogate neural network models of multiscale cellular material performance in the inverse identification of structural patterns has not been investigated.

In the current work, multiscale cellular material designs with hollow, variable-section inner designs are investigated. In particular, metamaterial designs with inner elements that are both hollow and follow a variable cross-sectional profile are analyzed for the first time. For the analysis, extensive numerical finite element simulations are performed, corroborated by selective experimental testing and analytical modeling results. It is shown that appropriate tuning of the second innermost scale can allow for the creation of lightweight, multiscale metamaterials, with enhanced normal, shear and bulk properties (Section 4). Furthermore, the necessary specifications for a neural-network-based association of the multi-scale input design parameters, with a complete set of constitutive and relative density effective properties, are identified. The high fidelity and low computation of the neural network model are highlighted, along with its potential coupling with inverse engineering analysis methods (Section 5).

## 2. Multiscale Hollow and Variable Inner Cross-Section Cellular Material Designs

### 2.1. Analytical and Numerical Characterization

**Figure 2:**
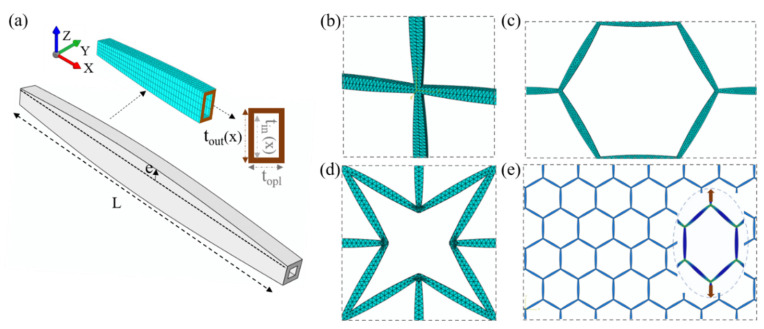
Hollow, variable-section element cross sectional attributes and swelling-to-length e/L characteristics (**a**). Finite-element unit-cell square (**b**), honeycomb (**c**) and re-entrant star-shaped (**d**) variable-section element lattices with $t_{o}=t_{opl}=1$ mm, e = 0.5 mm and an element length L of 20 mm. Periodic multiscale honeycomb FEM with a uniaxially loaded deformed cell is provided in (**e**).

We consider multiscale lattice material architectures composed of elements with a hollow variable-section inner form. The elements have outer and inner cross-sectional thickness $t_{o}$ and $t_{i}$, respectively, at their ends, as schematically depicted in Figure 2a. Both the outer- and inner-element thickness vary along the element length L, following a sinusoidal, half-period wave-form evolution of magnitude e—here named swelling—at the middle of the element (Figure 2a). The element’s area $A\left(x\right)$ and moment of inertia $I_{e}\left(x\right)$, thus, vary along the element length, and their evolution is defined as follows:

(1) $$\begin{array}{l} t_{out}\left(x\right)=t_{o}+2\;e\;sin\left[\frac{\pi x}{L}\right],\;t_{in}\left(x\right)=t_{i}+2\;e\;sin\left[\frac{\pi x}{L}\right],\;\left\{t_{o}>t_{i},\;0\leq x\leq L\right\},\\ A\left(x\right)=t_{out}\left(x\right)\;t_{opl}-t_{in}\left(x\right)\;t_{opl}^{v},\\ I_{e}\left(x\right)=I_{o}-I_{v},\;I_{o}=t_{opl}\;t_{out}{\left(x\right)}^{3}/12,\;I_{v}=t_{opl}^{v}\;t_{in}{\left(x\right)}^{3}/12 \end{array}$$

where $t_{opl}$ and $t_{opl}^{v}$ in Equation (1) stand for the out-of-plane total and hollow thickness parts, respectively (Figure 2a), while $sin\left[\pi x/L\right]$ characterizes the sinusoidal strut cross-section evolution. The hollow variable-section element form leads to a modification of the inner mass distribution, which depends on the swelling e and the inner-to-outer element thickness $t_{i}/t_{o}$ selection. Using as a reference the prismatic element case with a volume $V_{p}=t_{o}\;t_{opl}\;L$, the element-scale density modification $\rho_{e}^{*}$ is defined as:

(2) $$\begin{array}{c} \rho_{e}^{*}=\frac{\int_{0}^{L}A\left(x\right)dx}{V_{p}}=\frac{\pi \left(t_{o}t_{opl}-t_{opl}^{v}t_{i}\right)+4e\left(t_{opl}-t_{opl}^{v}\right)t_{i}}{\pi t_{o}t_{opl}},\\ \rho^{*}=\rho_{S_{2}}^{*}\rho_{S_{1}}^{*}=\rho_{e}^{*}\rho_{S_{1}}^{*} \end{array}$$

where $\rho_{e}^{*}$ in Equation (2) simplifies to unity for the prismatic, non-hollow case with zero swelling e, while the expression is independent of the element length L. The effective relative density $\rho^{*}$ at the metamaterial macroscale is determined by the relative density of the primal lattice design $\rho_{S_{1}}^{*}$, accounting for the second-scale density modification $\rho_{e}^{*}$. In Figure 2, different multiscale, hollow variable-section square (b), honeycomb (c) and star-shaped re-entrant (d) designs are depicted. The first-scale star-shaped lattice patterns refer to the auxetic lattice variant [17] and not to petal-form architecture variants, as investigated in [62].

For the computation of the effective multiscale metamaterial properties, two equivalent but distinct methodologies are followed. In the first case, multi-scale periodic finite element Abaqus models are created with a relative density of 0.03, 0.04 and 0.05 and an e value of 0.5, with element length values of 24, 30 and 40 mm and a $t_{i}/t_{o}$ of 0.5. The inner metamaterial structure is discretized with tetrahedral C3D10 solids using a fine mesh of more than 20 elements per inner constituent. For each of the lattice multiscale patterns (Figure 2e), a total of two independent loading cases, namely of a uniaxial tensile and shear load, suffice to compute the effective normal, shear and bulk properties. We note that all multiscale patterns investigated fall within the tetragonal symmetry space, with equal $E_{x}$ and $E_{y}$ moduli, as well as equal Poisson’s ratio values $\nu_{xy}$ and $\nu_{yx}$. The mechanical properties are obtained through the application of infinitesimal normal and shear strains $\varepsilon_{x}$ and $\gamma_{xy}$ of 0.001.

(3) $$E_{x}=\sigma_{x}/\varepsilon_{x},\;\nu_{xy}=-\varepsilon_{y}/\varepsilon_{x},\;G_{xy}=\sigma_{xy}/\gamma_{xy}$$

For periodic models with more than five unit-cell repetitions in each material direction, more than 60,000 elements are required for a single design case for convergent analysis results. The multiscale nature of the geometry poses practical analysis limitations in the parametric investigation of a wide range of inner multiscale topologies, and thus, different inner geometries and lattice configurations. This, in turn, limits the dataset size that can be created, an important parameter for the neural network analysis part elaborated in Section 3.

In the second methodological modeling approach, the previous limitation is surpassed, applying a two-scale homogenization process. Initially, the homogenized axial and bending stiffness of the inner variable-section element geometry are computed as a function of geometric and material properties ($t_{o}$, $t_{i}$, L, e, $E_{s}$) for the innermost scale $S_{2}$ (Equation (1), Figure 1). Thereafter, the macroscale lattice’s effective attributes are obtained based on the homogenized second-scale $S_{2}$ properties, which are employed as inputs for the first-scale $S_{1}$ computations, making use of the homogenization algorithm elaborated upon in [32]. The effective metamaterial properties (E, K, G, ν) are a direct result of the asymptotic homogenization, which computes the complete flexibility and stiffness matrix tensor of a given multiscale periodic pattern, provided with the homogenized normal and bending stiffness attributes of its inner constituents. The reader is referred to [32] for a detailed description of the theoretical formulation, as well as for its numerical implementation. The previously explicated algorithmic process allows for the parametric computation of a wide range of multiscale cellular configurations.

For the computation of the homogenized $S_{2}$ element-scale stiffness attributes, analytical formulas for the effective axial $k_{N}^{e}$ and bending $k_{B}^{e}$ stiffness attributes are derived, using the unit-force method. The corresponding analytical mechanics expressions contain integral forms with varying area and moment-of-inertia properties (Equation (1)). In particular, the axial flexibility $f_{N}^{e}$ is computed as $\int_{0}^{L}\left(N\cdot \bar{N}\right)/\left(EA\left(x\right)\right)dx$ − $\bar{N}$ being the virtual axial force inner element evolution created by a unit normal axial load at the element ends -, while the bending flexibility $f_{B}^{e}$ is computed as $\int_{0}^{L}\left(M\cdot \bar{M}\right)/\left(EI_{e}\left(x\right)\right)dx$ − $\bar{M}$ being the virtual bending moment developed within a clamped beam element, subsect to a shear type forces at its ends -. The corresponding stiffness terms are obtained by computing the inverse of the flexibility components, $k_{N}^{e}=1/f_{N}^{e}$ and $k_{B}^{e}=1/f_{B}^{e}$ accordingly. The reader is referred to [27,63] for a detailed description of the virtual force method.

### 2.2. Additive Manufacturing and Experimental Characterization

**Figure 3:**
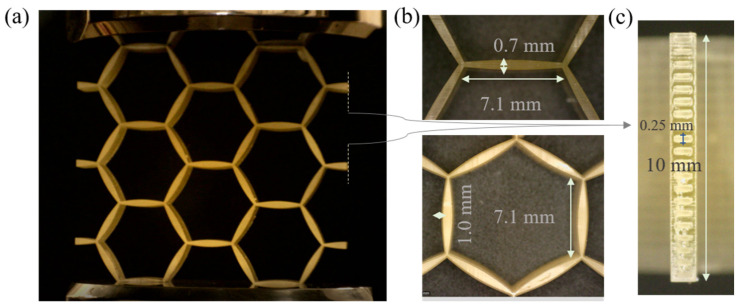
Experimental testing of micro-fabricated multiscale honeycomb lattices (**a**). Elements with a length of 7.1 mm and different inner element geometries are depicted in (**b**). A 10 mm out-of-plane thickness with a hollow-width feature size of 0.25 mm is depicted in (**c**), rotated by 90° with respect to (**a**).

The elastic stiffness of two-scale honeycomb metamaterial designs was experimentally investigated using 3D-printed periodic specimens (Figure 3a). Two distinct, multiscale honeycomb designs were fabricated with an element length L of 7.1 mm, an out-of-plane thickness of 10 mm (Figure 3b,c) and different inner-element hollow profiles. In particular, element profiles with an e value of 0.17 and 0.36 mm were fabricated, with the corresponding honeycomb periodic designs named D1 and D2. The $L/t_{o}$ ratio was set, in all design cases, as equal to 18, while the $t_{opl}/t_{o}$ parameter was set to 0.5. For the fabrication, a BMF 3D printer was employed with a high-temperature resin. For each honeycomb design, three repeat samples were conducted. A total fabrication time of several tenths of hours was required for the 3D printing of the specimens, noting the substantially high number of slices necessary for a sufficiently accurate geometric representation. The multiscale honeycomb structures consisted of a total of 20 periodic cells stacked in the out-of-plane thickness direction (Figure 3c); the stacking was employed to ensure sufficient stiffness and surface area along the specimen thickness direction for the load application upon mechanical testing.

For the testing, an Instron machine with a 50 N loadcell was employed. A rather low testing speed of 1 mm/min was used, corresponding to a strain rate below 0.01/s to ensure static loading conditions for all specimens.

## 3. Machine-Learning-Based Modeling and Design of Multiscale Metamaterial Architectures

Effective macroscale metamaterial properties were determined by a substantial number of inner design parameters. Even for a given base material ($E_{s}$) and first-scale $S_{1}$ unit-cell pattern (e.g., honeycomb, square, Figure 2), the metamaterial attributes were a function of the element thickness $t_{o}$ and length L, as well as the inner-element hollow thickness $t_{i}$ and swelling parameter e. For a single combination of the previously reported parameters, a set of a minimum of two independent computational tasks needed to be performed for the effective normal moduli E, bulk K, shear G and Poisson’s value ν to be obtained. More importantly, no explicit functions associating the effective macroscale properties with the inner first $S_{1}$ and second $S_{2}$ scale design parameters (Figure 1) were available, so that multiscale inverse engineering tasks could not be performed.

For the modeling of the mechanical response of a wide range of inner-material designs, a machine-learning-based approach, without prior assumptions with respect to the observed macroscale constitutive metamaterial response, was followed. Five primal inner design parameters were used as input features (I), namely two first-scale ($S_{1}:$ $t_{o},\;L$) and two second-scale ($S_{2}:\;t_{i},\;e$) attributes, as well as the base material modulus $E_{s}$. For the current analysis, a unit out-of-plane thickness $t_{opl}$, along with a half unit $t_{opl}^{v}$ out-of-plane void thickness part, were considered (Figure 2a), without loss of generality. The neural network output parameters (O) included the complete set of the elastic E, bulk K and shear G values, as well as the Poisson’s ratio value ν (Figure 1) and relative density $\rho^{*}$ data, computed using multiscale homogenization (Section 2.1). The neural network model can be viewed as a multivariate regressor of the mechanical performance. We note that all multiscale patterns investigated fell within the tetragonal symmetry space, so that equal $E_{x}$ and $E_{y}$ moduli and Poisson’s ratio values $\nu_{xy}$ and $\nu_{yx}$ applied, allowing for the simplification of the corresponding notations.

For each unit-cell primal design case, several effective metamaterial performance data in each feature direction were created. In particular, eleven data points along the element thickness $t_{o}$, seven data points along the inner hollow element thickness $t_{i}$ and element length L, and eleven data points along the swelling e feature direction were employed. The data points were created in a hierarchical manner, so that for a given element thickness $t_{o}$ and base material moduli $E_{s}$ at the first cellular scale $S_{1}$, normalized slenderness and second-scale feature attributes were created ($t_{i}$/$t_{o}$, $t_{o}$/L, e/$t_{o}$), covering all possible parameter combinations (summarized in Table 1). For each feature, uniform spacing among the indicated bounds was applied for simplicity. It is noted that the bounds prescribed covered a wide range of inner cellular designs, albeit non-exhaustive with respect to the possible design space of multiscale metamaterial patterns. The number of sampling points for each parameter was selected based on initial reduced input-dimensionality-fitting studies; they were defined so as to allow for high-accuracy results, retaining an overall low data-creation computational cost. Moreover, it should be underlined that the multiscale metamaterial performance is affine with respect to the base material modulus $E_{s}$, so that it could be omitted from the modelling process, if all the results were to be obtained in a normalized, non-dimensional form. The effective metamaterial attributes for each multiscale design were computed using the two-scale homogenization process explicated at the end of Section 2.1.

**Figure 4:**
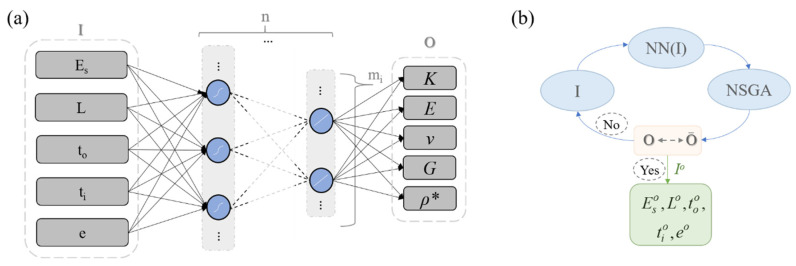
Machine learning modeling architecture for the prediction of the effective normal, bulk, shear, Poisson and relative density properties based on first- and second-scale inner design parameters (**a**). The model size defined by the number of layers n and neurons per layer m, as well as the activation function employed, constitute supervised learning parameters to be determined. The trained NN model is coupled to a Non-dominated Sorting Genetic Algorithm (NSGA) for the identification of optimal design parameter sets (**b**).

The parameter range of Table 1, along with the discretization introduced for each feature, leads to more than 53,000 metamaterial design cases for a given multiscale lattice pattern (Figure 1). The different unit-cell multiscale designs were created in a parametric manner, following the above elaborated process. The input features (I) of Table 1 are related to the macroscale effective metamaterial attributes (O) through a neural network model (Figure 4). The network design and computational complexity, as characterized by the number of layers n and neurons m per layer (Figure 4a) are parameters to be determined throughout the training process [64]. Neural network architectures with a minimum of two and up to five hidden layers were considered, with a minimum of 5 and up to 20 neurons per layer. A schematic of the machine modeling architecture is provided in Figure 4a.

For the training of the model, different activation functions were investigated, including the tanh, sigmoid, and deep learning common-purpose activation functions, such as the relu activation function [43]. For the training process, the mean squared error function was used (MSE), along with the Levenberg–Marquardt default optimization algorithm. The data were shuffled before training. The mean squared error was defined through the difference of the neural network (NN) from the multiscale homogenization (MH) mechanical parameters $MSE=\left(1/N\right){\sum_{N}\left(O^{MH}-{\bar{O}}^{NN}\right)}^{2}$. Throughout the training process, 30% of the available data created with the process previously explicated were used for testing. The trained NN models were independently controlled with respect to their accuracy, using an additional validation dataset of 1000 multiscale cellular designs, created through random input feature generation in a design space that exceeded the bounds set for each feature in Table 1 by up to 20%. The weighting matrix coefficients $W^{i}$, the layer constants $W_{0}^{i}$, and the layer activation function f and network depth n were parameters to be determined throughout the training process [64].

(4) $${\bar{O}}^{NN}=f^{n}\left(W^{n}f^{n-1}\left(\ldots W^{3}f^{2}\left(W^{2}f^{1}\left(W^{1}I+W_{0}^{1}\right)+W_{0}^{2}\right)\ldots \right)+W_{0}^{n}\right)$$

The trained model was used as a surrogate for its coupling with the genetic, multi-objective optimization algorithm (Non-dominated Sorting Genetic Algorithm, Figure 4b) elaborated in [65] for the identification of Pareto set solutions that satisfy both base material moduli and density targets at the metamaterial macroscale. For the inverse analysis, the neural network modeling parameters of Figure 4a were used as inputs, while the effective elastic modulus and relative density value were concurrently used as macroscale optimization objectives. For the generic algorithm computation, a probability of crossover and mutation of 0.9 and 0.5 were used accordingly, along with a mutation parameter of 0.05 [65]. The population size and number of generations required for convergence are discussed in Section 5.

## 4. Effective Mechanical Attributes of Multiscale, Variable Inner Section Cellular Materials

The variable-section, doubly sinusoidal variation of the inner geometry result in a non-linear area and moment of inertia distribution along the element length, as indicated by the function definitions of Equation (1). An analytical computation of the effective axial $k_{N}^{e}$ and bending $k_{B}^{e}$ stiffness at the element scale using the classical mechanics formulations summarized in Section 2.1 is infeasible with the use of commercial integration routines (Mathematica 11.3, Maple 2020), retaining the sinusoidal geometric definitions of Equation (1). However, by employing the Bhaskara approximation of the $\sin\left(x\right)$ function [66] (Appendix A), closed-form expressions of the element’s effective axial and bending stiffness attributes are obtained, as follows:

(5) $$\begin{array}{ll} k_{N}^{e}= & E_{s}/\left(\frac{L}{8e\left(t_{opl}^{v}-t_{o}\right)-t_{opl}^{v}t_{i}+t_{o}t_{opl}}+\frac{20eL\left(t_{opl}-t_{opl}^{v}\right)a\coth\left(c1\right)}{c_{2}^{3/2}c_{3}}\right),\\ k_{B}^{e}= & E_{s}t_{opl}/\left(L^{3}\left(\frac{t_{o}^{3}+c_{4}}{{\left(t_{o}-8e\right)}^{4}{\left(t_{o}+2e\right)}^{2}}+\frac{c_{5}}{c_{6}}\right)\right)\\ & -E_{s}t_{opl}^{v}/\left(L^{3}\left(\frac{t_{i}^{3}+c_{7}}{{\left(t_{i}-8e\right)}^{4}{\left(t_{i}+2e\right)}^{2}}+\frac{c_{8}}{c_{9}}\right)\right) \end{array}$$

where αcoth in Equation (5) stands for the inverse hyperbolic cotangent function. The constants $c_{1}-c_{9}$ entering the expressions of Equation (5) are detailed in Appendix A. For zero swelling *e*, the effective axial stiffness $k_{N}^{e}$ simplifies to the well-known axial stiffness form of a prismatic element cross-sectional profile. The same applies to the bending element stiffness $k_{B}^{e}$ for which the constants $c_{4},\;c_{5},\;c_{7}$ and $c_{8}$ vanish.

**Figure 5:**
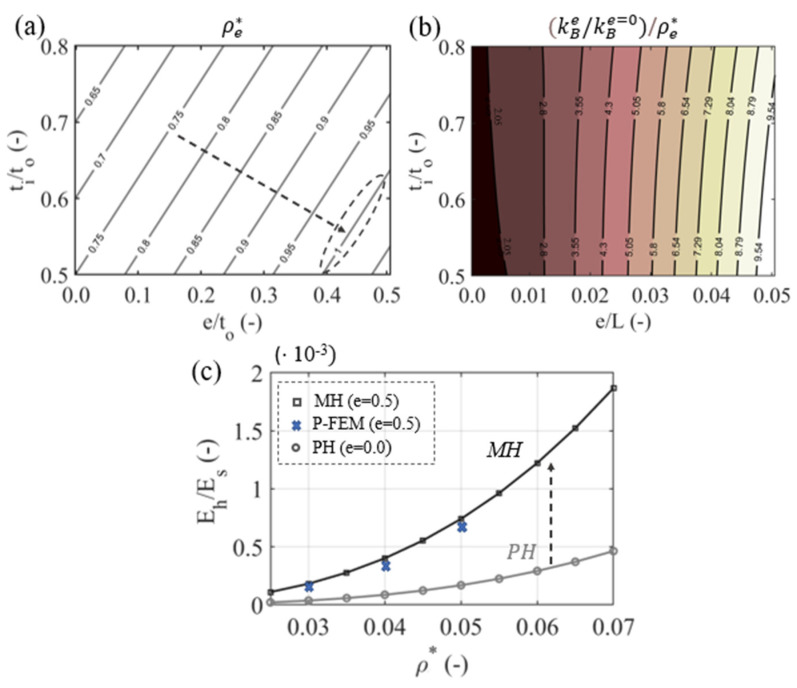
Dependence of the relative density of hollow variable-section elements on the swelling-to-end-element-thickness ratio $e/t_{o}$ and on the $t_{i}/t_{o}$ ratio (**a**). The enhancement of the inner specific bending stiffness (normalized to $\rho_{e}^{*}$ of Equation (2) and to the prismatic bending stiffness element case) as a function of the swelling-to-length e/L and inner-to-outer-element-thickness $t_{i}/t_{o}$ is provided in (**b**). A length L = 20 and a $t_{o}$, $t_{opl}$ and $t_{opl}^{v}$ of 1, 1 and 0.5 mm are used, respectively. The effective elastic modulus scaling with relative density for a multiscale honeycomb lattice (e = 0.5) and a prismatic honeycomb (e = 0.0) is depicted in (**c**).

In Figure 5a, the scaling of the relative density as a function of the element geometry $e/t_{o}$ and cross section hollowness $t_{i}/t_{o}$ is provided. The effect of the variable inner-element section design on the specific bending-element stiffness is illustrated in Figure 5b as a function of the swelling-to-length ratio e/L. The modified inner (S_2_, Figure 1) normal and bending stiffness attributes can lead to a different density scaling of the effective elastic macroscale cellular properties. In Figure 5c, the scaling law of the effective modulus with respect to the relative density ($E/E_{s}=A\rho_{}^{{*}^{n}}$) of variable-section, multiscale honeycombs (MH), with a swelling parameter value e = 0.5, is provided, along with the case of prismatic, single-scale honeycombs (PH). The two-scale MH mechanical properties are computed using the multiscale homogenization process from Section 2.1. The periodic finite-element modeling results (P-FEM, Figure 2e) are depicted by blue cross symbols.

The relative density of the hollow variable-section geometry is below unity over a wide range of variable-section hollow element forms (Figure 5a), allowing for lightweight material designs. More importantly, the specific bending stiffness of the variable-section inner geometry is enhanced (Figure 5b), with rather small e values to yield substantial stiffness improvements compared to the prismatic design case. It is noted that the inner stiffness enhancement is strongly non-linear. Doubling the maximum swelling e increases the specific bending stiffness more than two times (Figure 5b).

The improved second-scale material performance can modify the scaling of the effective macroscale elastic modulus E with respect to the metamaterial relative density $\rho^{*}$. In particular, the least square fitting for the PH case yields A and n coefficients of 1.3 and 3, respectively, which is in agreement with the theoretical predictions [20] (e = 0, data from Table 1). For the MH case with e = 0.5, the A coefficient more than doubles to 2.8 compared to the theoretical prediction of 1.3 for the PH case, while the corresponding density exponent n is reduced to 2.75. 

**Figure 6:**
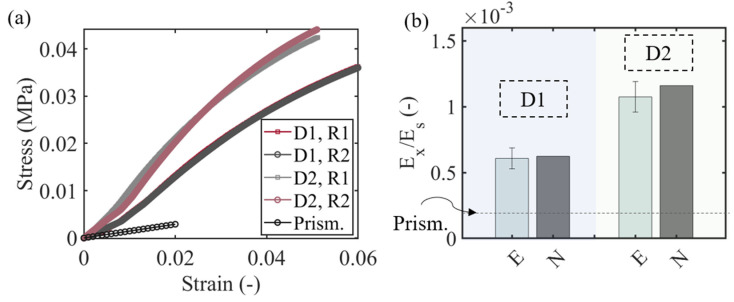
Experimental stress-strain response of multiscale honeycombs with two distinct inner element profiles, along with the reference prismatic design case (**a**). Relative stiffness increases with respect to the prismatic, single-scale configuration for each of the multiscale, variable-section honeycomb design cases -experimental (E) and numerical (N) results (**b**).

In Figure 6a, the experimentally obtained stress–strain results for the D1 and D2 design cases of Section 2.2 are provided, along with the prismatic reference case. The experimentally obtained (E) elastic stiffness for each design case is depicted in Figure 6b—computed in the low-strain range (<1%)—along with the multiscale homogenization numerical analysis results (N). For each design case, two distinct experimental repeats are presented (R1, R2 in Figure 6).

The experimental results of Figure 6a indicate a clear elastic stiffness increase with higher swelling parameter e values (Figure 6a), with the highest swelling D2 design case considerably outperforming the stiffness of the reference prismatic design. The stiffer response is retained throughout the entire strain range investigated. It should be noted that the enhanced constitutive performance can be obtained at a practically invariant relative density value of the effective metamaterial, noting the near unity $\rho_{e}^{*}$ value corresponding to the design specifications of the multiscale honeycomb designs D1 (Figure 6a). The elastic stiffness of the moderate e design case D1 is more than three times higher the prismatic, single-scale design case, a performance-improvement scaling factor that exceeds 7 for the high e D2 design case (Figure 6b). The experimental data lie in good accordance with the numerical predictions for both density cases tested, verifying the substantial difference of the MH case with respect to the PH single-scale designs. It should be noted that the stiffness increase effect observed upon the use of variable-section hollow inner-strut designs does not apply to all first-scale cellular patterns, as explicated in the sequel.

**Figure 7:**
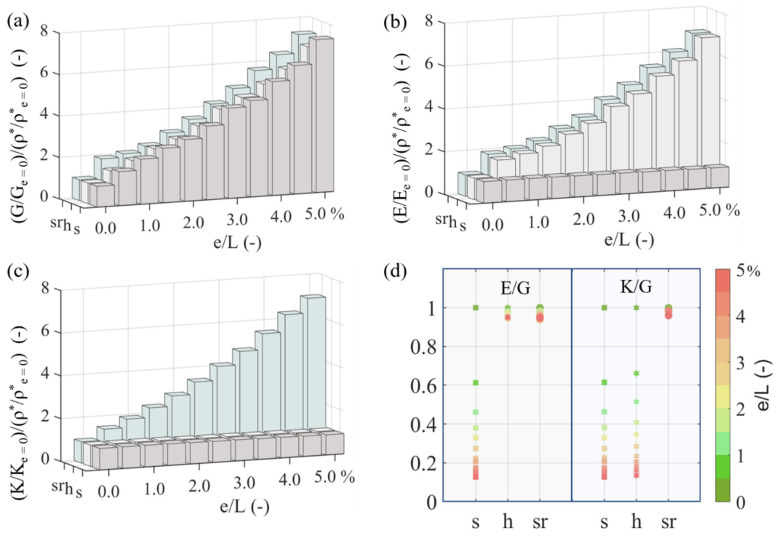
Specific shear (**a**), normal (**b**) and bulk (**c**) modulus per relative density evolution of square (s), honeycomb (h) and star re-entrant (sr) lattice patterns for different element-scale ratios e/L. The specific normal-to-shear E/G and bulk-to-shear K/G moduli ratio values are provided in (**d**) over the same range of e/L values. The analysis is performed for a $L/t_{o}$ ratio of 20 and a $t_{o}$ and $t_{i}$ of 1 and 0.8 mm, respectively.

In Figure 7, the effect of the inner second-scale on the macroscale elastic E, shear G and bulk K material properties of multiscale square (s)-, honeycomb (h)- and star re-entrant (sr)-shaped cellular patterns is analyzed in a comparative manner. In particular, not only the specific elastic normal modulus, but also the specific shear and bulk modulus (Figure 7a–c) are given for different swelling-to-length e/L values. The mechanical properties are normalized with respect to the prismatic design reference case. All computations pertain to an $L/t_{o}$ of 20 with a $t_{i}/t_{o}$ of 0.8 and a $t_{o}$ of 1 mm. Moreover, a unit out-of-plane thickness $t_{opl}$, along with a half-unit $t_{opl}^{v}$ out-of-plane void thickness part, is considered. The computations are performed following the multiscale homogenization process elaborated upon in Section 2.1. In Figure 7d, the evolutions of the normal-to-shear E/G and bulk-to-shear K/G moduli ratios over the same range of e/L values are provided.

The employment of hollow, variable-section inner designs at the second inner scale (S_2_) of the cellular material leads to an increased specific metamaterial shear stiffness for all lattice patterns in Figure 2. More specifically, the specific shear stiffness enhancement is up to approximately seven times the shear resistance of the reference hollow lattice pattern for an e/L value of 5%, with the increase being non-linear with respect to the swelling-to-length parameter. An analogous improvement is recorded for the axial modulus $\left(E/E_{e=0}\right)/\left(\rho^{*}/\rho_{e=0}^{*}\right)$ of the honeycomb and square re-entrant lattice patterns, with the specific axial modulus of the square lattice remaining practically unaffected (Figure 4b). Accordingly, the specific bulk modulus $\left(K/K_{e=0}\right)/\left(\rho^{*}/\rho_{e=0}^{*}\right)$ of the star re-entrant metamaterial design increases, contrary to the bulk resistance of the square and honeycomb lattice patterns, which remain practically invariant.

The multiscale designs yield a macroscale cellular material response (Figure 7a–c) that differs for each of the first-scale lattice patterns ($S_{1}$), so that the material design space extension induced by the innermost architectural scale ($S_{2}$) is non-unique. More specifically, the relative performance of the multiscale, variable-section metamaterial designs to normal loads and shear loads (E/G) remains practically unaffected for the honeycomb and re-entrant square lattice patterns (Figure 7d), contrary to the square lattice case, where a shear-stiffer response is observed (Figure 7d). Accordingly, the bulk-to-shear effective metamaterial performance (K/G) becomes shear-stiffer for the square and honeycomb lattice case, remaining unaffected for the re-entrant square lattice pattern.

## 5. Neural-Network-Based Multiscale Metamaterial Forward Modeling and Inverse Design

**Figure 8:**
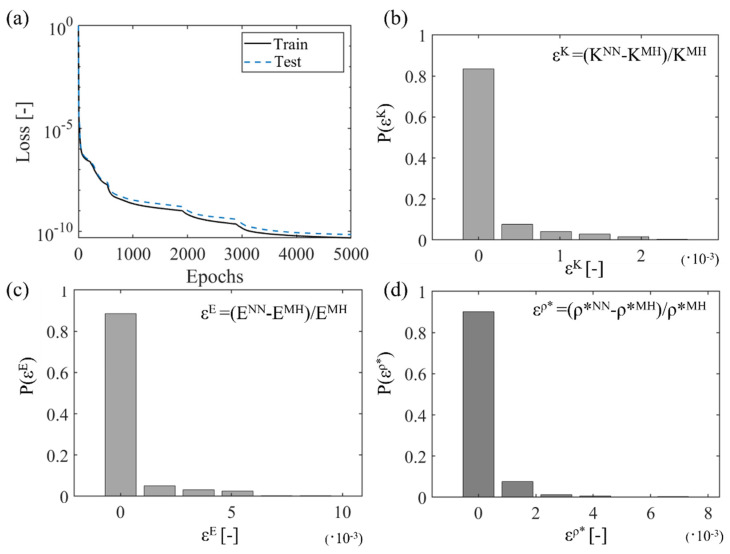
Training and test loss curves for the neural-network (NN)-based modeling of the mechanical response of multiscale honeycomb metamaterial designs (**a**). Error probability distribution of the bulk (**b**) and normal (**c**) modulus and relative density value (**d**) for the validation dataset of 1000 random-input-feature generation. Relative error values below 0.05% are considered, for all practical purposes, as zero.

Multiscale metamaterial performance is determined by several first- and second-scale parameters, as well as by the base material modulus $E_{s}$. To predict the complete set of effective metamaterial properties, as summarized in Figure 4a, different depth neural network architectures were probed. It was observed that a highly accurate mapping of the input design features (I) with the output effective material properties (O) is feasible for all activation functions listed in Section 3. However, networks using the ReLu activation function require more than double the number of training parameters and deeper network architecture than the corresponding sigmoid or hyperbolic tangent counterparts for the same accuracy level to be achieved. Networks with a total number of four layers and less than one thousand training parameters were observed to provide the highest modeling accuracies, as quantified by the training process. The minimum testing loss performance was obtained for networks with 20 × 15 × 5 × 5, 16 × 10 × 5 × 4 and 16 × 12 × 6 × 5 neurons for the multiscale honeycomb, square and re-entrant square cellular patterns. All network architectures are provided in the form of supplementary material. Networks with higher computing cost and comparable loss performance were excluded from the analysis. In the independent validation sets used for each of the multiscale cellular patterns here investigated, a relative error among the multiscale homogenization and the NN predictions below 1% for all QoI of Figure 4b was recorded. In Figure 8a, insights in the training and testing loss curves for the case of a multiscale honeycomb lattice over a training period of 5000 epochs are provided. Figure 8b–d depict frequency error bars for the bulk (K) and normal (E) modulus and relative density neural network predictions for the validation dataset of the 1000 randomly generated multiscale cellular designs (Section 3). For the relative error estimation, the multiscale homogenization (MH) results are used as a reference for the NN prediction comparisons.

The loss curves of Figure 8a reveal a multi-level training process, which spans several orders of magnitude of performance improvement over 5000 epochs. The trained neural network model can predict the effective multiscale properties with substantial accuracy comparable to state-of-the-art relevant modeling contributions [40] over the entire space, as indicated from the loss curve in Figure 8a. The error frequency distribution for the bulk, elastic and relative density predictions denotes a relative material performance prediction error that is substantially lower than 1% for all NN model predictions (Figure 8b–d). It should be noted that the sharp variation in the loss function in Figure 8a has to be associated—to a certain extent—with the incorporation of the base material modulus $E_{s}$ in a dimensional form in the input neural network model parameters (Section 3). All trained neural network architectures are provided in the form of complementary material for completeness purposes.

**Figure 9:**
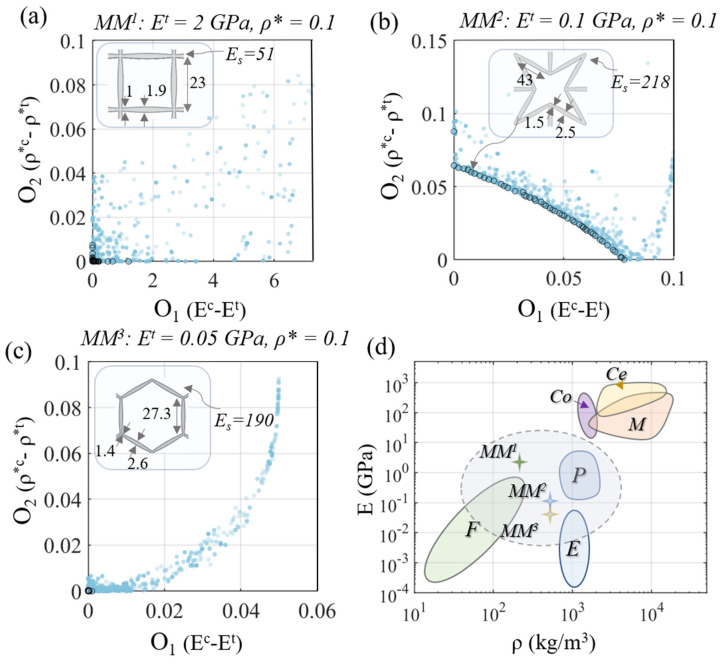
Optimal Pareto front for a multiscale square lattice with a target modulus of E^t^ = 2 GPa and a relative density of 0.1 (**a**). The optimal pareto front solution for a multiscale square re-entrant and multiscale honeycomb cellular with a target modulus of E^t^ = 0.1 GPa and E^t^ = 0.05 GPa and a relative density of 0.1 are provided in (**b**) and (**c**), respectively. Inversely identified structural multiscale cellular patterns are depicted in each case. The metamaterial (MM) effective modulus is presented in an Ashby diagram comparative form in (**d**). For completeness, the modulus-density ranges for foams (F), elastomers (E), polymers (P), metals (M), composites (Co) and ceramics (Ce) are included.

The elaborated NN-based metamaterial models provide a direct link of the inner scales (Figure 4a) with the complete set of elastic effective metamaterial attributes. As a result, inverse multiparametric identification tasks become feasible. In particular, given a set of macroscale material performance objectives, the inner design specifications that best match the prescribed criteria can be probed. We note that the inverse structural identification analysis conducted herein is restrained within the mechanical performance limits of the multiscale cellular patterns investigated. As such, its scope and functionality need to be clearly separated from free-morphology or full-topology optimization methods [49,51], which are beyond the analysis range and context of the current contribution. Using the genetic algorithm elaborated upon in [65], the base material modulus $E_{s}$, along with the second $S_{2}$ and first $S_{1}$ material scale features required for a multiscale lattice pattern, to yield a desirable macroscale performance, can be identified. In Figure 9a,b, the evolution of the Pareto front for a target elastic modulus O_1_ and relative density O_2_ objective are provided. A target modulus $E^{t}$ of 2 GPa and 0.1 GPa, with relative densities $\rho^{*}$ of 0.1 (O_2_, Figure 9a,b), respectively, are employed for a multiscale square and square reentrant design. Accordingly, the evolution of the Pareto front solution for the case of a multiscale honeycomb lattice pattern is provided in Figure 9c. For simplicity, the hollow element thickness is set to be fifty percent of the outer-end element thickness $t_{o}$ in all computations. One of the best sorting structural patterns is provided in each case, along with the evolution of the optimal Pareto front solution (using a population size of 30 and 150 generations). In Figure 9d, the identified optimal effective material modulus is given in the form of an Ashby diagram, while the base material modulus required for each target metamaterial performance is provided in Figure 9a–c.

The results of Figure 9 provide insights into the inverse engineering potential of the neural network multiscale metamaterial models developed. More specifically, given the high accuracy and the low computational cost of a single model evaluation, multi-objective, genetic-algorithm-based computations, requiring thousands of model evaluations are feasible in a few seconds. By that means, the base material modulus $E_{s}$ and the geometric features of the second ($S_{2}$) and first ($S_{1}$) design scale can be probed to optimally meet the macroscale material objectives (Figure 9). Moreover, the potential of a given multiscale cellular material to meet a set of macroscale objectives (O) can be robustly assessed, providing quantitative estimates of its optimality in each material performance direction. This becomes explicitly evident in Figure 9b, where none of the objectives can be fully satisfied. However, the Pareto front solutions allow for the inverse identification of inner multiscale cellular patterns that either fully satisfy one of the objectives, or partially satisfy each, quantifying the relative performance discrepancies. What is more, the inferred base-material moduli values $E_{s}$ can be directly used to assess the manufacturing feasibility of the metamaterial part, confining the base-material selection range and 3D printing technologies that could be employed in the fabrication process.

## 6. Discussion

While it is well established that multiscale metamaterial architectures can provide additional degrees of freedom for design that can extend the performance limits of single-scale patterns, the corresponding bounds remain, to a great extent, unquantified. The employment of hollow variable-section elements provides the possibility to modulate the normal- and bending-stiffness attributes of the inner lattice constituents. The stiffness alteration is controlled by the variable-section inner geometry. Element profiles with a rather small variation in their inner-thickness-to-length ratio (e/L) allow for considerable bending stiffness enhancements (Section 2.1). The elaborated analytical stiffness forms indicate that the bending performance can be tuned depending on the inner length L and on the element profile geometric swelling parameter e. However, it can alter the reference bending stiffness performance by up to one order of magnitude for rather low e/L values, in a controlled manner (Figure 5b).

The multiscale designs modify the macroscale metamaterial performance in a non-linear manner, which depends on both the first-scale $S_{1}$ pattern and on the second-scale design. In particular, while a specific shear stiffness increase is recorded for all the multiscale lattice patterns in Figure 7a (rs, s, h), denoting a shear strengthening per unit weight for all design cases, different behaviors with respect to the normal and bulk metamaterial attributes are observed. More specifically, variable-section multiscale honeycombs allow for increased normal moduli with an invariable specific bulk modulus (Figure 7), while multiscale square patterns retain invariance both in their specific bulk and normal stiffness characteristics. This observation indicates that the resistance of certain multiscale metamaterial designs to shape changes can be controlled through appropriate modifications of the second, innermost scale, with volume changes favored over shape changes. Moreover, there exist first-scale ($S_{1}$) lattice designs for which a simultaneous increase in the specific normal, shear and bulk stiffness is induced; this is the case for multiscale square-re-entrant patterns (Figure 7), with the relative volumetric and shape metamaterial resistance remaining practically invariable (Figure 7d).

The decoupled second- and first-scale geometry allows for the preservation of the connectivity of the upper-scale $S_{1}$ lattice pattern. All the multiscale lattice cases investigated above have a nodal connectivity that is less than 6, so that the bending stiffness of their inner constituents is significant [67,68], for the metamaterial’s macroscale performance. Multiscale designs of this kind can yield enhanced density-scaling laws, which substantially differ from the single-scale metamaterial architecture, as demonstrated for the multiscale honeycomb case (Figure 5c).

However, the estimation of a complete set of effective mechanical properties can be a substantially cumbersome process due to the different scales and the number of parameters involved. The results of Section 5 indicate that a direct link between the inner multiscale geometric and material design attributes and the macroscale metamaterial performance is feasible, with the use of appropriately architected neural network models. In particular, rather low-computational-cost neural network architectures have been identified that can robustly predict the complete set of effective elastic properties, including the bulk and shear metamaterial response. The accuracy of the networks is in the third decimal order for all quantities of interest (Figure 8), with the model execution time amounting to some fractions of a second on an ordinary personal computing system.

The low computing cost of the neural network models elaborated allows for their coupling with inverse, genetic-algorithm-based, multi-objective optimization schemes (Figure 9) in a single-step process. By that means, a wide range of optimization engineering tasks can be performed. In particular, metamaterial designs that can optimally satisfy a combination of macroscale performance objectives can be inversely identified in the form of Pareto front solutions. This allows for the rigorous assessment of the engineering feasibility of a certain design; moreover, it allows for the identification of solution sets that can concurrently best meet performance requests within a given multiscale metamaterial class, as demonstrated in Figure 9b. In the inverse identification process, both the geometric and the base material modulus requirements are identified with the necessary moduli specifications and can be directly probed.

## 7. Conclusions

Overall, the current work investigated multiscale variable-element-section cellular metamaterial designs, combining analytical, numerical and experimental testing methods. Analytical expressions for the effect of variable-section, hollow element designs on the inner-axial and bending strut-scale stiffness were elaborated. The mechanical performance of a wide range of multiscale cellular designs were evaluated. It was observed that:

1. Hollow, variable-section inner structural designs allow for an enhanced, specific normal, shear and bulk metamaterial response, well beyond the range of single-scale metamaterial architectures.
2. The insertion of a second inner scale affects the macroscale metamaterial performance in a non-unique manner, which depends on the uppermost-scale cellular pattern design.
3. Multiscale designs can modify the stiffness-to-density scaling of cellular materials from a bending-dominated towards a stretching-dominated response.
4. Low-numerical-cost neural network models can derive a robust link between the different inner scales and the complete set of effective elastic cellular material properties.
5. Inverse multi-objective engineering tasks can, therefore, be performed, identifying the optimal multiscale cellular patterns that best satisfy the macroscale performance requests.

We hope that the methodology elaborated and the results provided act as a framework in the analysis and design of multiscale cellular materials beyond the design cases investigated herein.

## Figures and Tables

**Table 1:** First-scale $S_{1}$ and second-scale $S_{2}$ metamaterial design parameter range used for the creation of training datasets for multiscale square, honeycomb and star re-entrant lattice patterns.

Input Features	Range	Sampling Points
E_s_	50–210 (GPa)	9
$t_{o}$	0.5–1.5 (mm)	11
$t_{i}/t_{o}$	0.2–0.8 (-)	7
$t_{o}/L$	1/20–1/50 (0)	7
$e/t_{o}$	0–1 (-)	11

## Appendix A

The approximation of the sin(x) function entering the geometric definitions of Equation (A1) is given by the Bhaskara formula, as follows [66]:

(A1) $$\sin\left(x\right)=16x\left(\pi -x\right)/\left(5\pi^{2}-4x\left(\pi -x\right)\right)$$

With the use of the Bhaskara approximation of Equation (A1), the constants $c_{1}-c_{3}$ entering the analytical stiffness computations are computed as a function of $t_{o},\;t_{i},\;t_{opl},\;t_{opl}^{v}$ and of the element swelling parameter e, defined in Figure 1. For the axial stiffness case, we obtain:

(A2) $$\begin{array}{l} c_{1}=2\sqrt{t_{opl}^{v}\left(2e+t_{i}\right)-t_{opl}\left(2e+t_{o}\right)}/\sqrt{8e\left(t_{opl}^{v}-t_{opl}\right)-t_{opl}^{v}t_{i}+t_{opl}t_{o}}\\ c_{2}=8e\left(t_{opl}^{v}-t_{opl}\right)-t_{opl}^{v}t_{i}+t_{opl}t_{o},\;c_{3}=\sqrt{t_{opl}^{v}\left(2e+t_{i}\right)-t_{opl}\left(2e+t_{o}\right)} \end{array}$$

Accordingly, the constants $c_{4}$ to $c_{6}$, entering the stiffness definitions of Equation (5), are given as follows:

(A3) $$\begin{array}{l} c_{4}=-3572e^{3}-3268e^{2}t_{o}-364et_{o}^{2}+360e{\left(2e+t_{o}\right)}^{2}\left(\log\left[5L^{2}t_{o}\right]-\log\left[4L^{2}\left(2e+t_{o}\right)\right]\right)\\ c_{5}=30\;e\cdot \left(784\;e^{3}+581\;e^{2}\;t_{o}+318\;e\;t_{o}^{2}+18\;t_{o}^{3}\right)a\coth\left[\frac{2\sqrt{2e+t_{o}}}{\sqrt{8e-t_{o}}}\right]\\ c_{6}={\left(-t_{o}+8e\right)}^{9/2}\cdot {\left(t_{o}+2e\right)}^{5/2} \end{array}$$

Accordingly, the constants $c_{7}$ to $c_{9}$, entering the stiffness definitions of Equation (5), are given as:

(A4) $$\begin{array}{l} c_{7}=-3572e^{3}-3268e^{2}t_{i}-364et_{i}^{2}+360e{\left(2e+t_{i}\right)}^{2}\left(\log\left[5L^{2}t_{i}\right]-\log\left[4L^{2}\left(2e+t_{i}\right)\right]\right)\\ c_{5}=30e\cdot \left(784e^{3}+581e^{2}t_{i}+318et_{i}^{2}+18t_{i}^{3}\right)a\coth\left[\frac{2\sqrt{2e+t_{i}}}{\sqrt{8e-t_{i}}}\right]\\ c_{6}={\left(-t_{i}+8e\right)}^{9/2}\cdot {\left(t_{i}+2e\right)}^{5/2} \end{array}$$
